# A Medical Officer of Health for Georgia

**Published:** 1896-10

**Authors:** 


### A MEDICAL OFFICER OF HEALTH FOR GEORGIA.

If one were asked to say what is the most useful branch of medical science, and would for the occasion lay aside a dislike to comparisons in such matters, one would emphatically reply, "Preventive Medicine," and would no doubt be telling the simple truth if reference were made to certain places. But for others this would scarcely hold good, because in them it is practically non-existent, and it is really true of only those districts in which the best possible work is being done in this connection. Preventive medicine, in a modern fulness of the word, is comparatively a young science; it has made immense and rapid strides; but it is only when we look into the vista of the future that we see it in the greatness which is its proper right.

Recent work shows more and more the tendency to look to prevention as the easiest and most potent method of ameliorating the suffering of mankind, for what else do all the novel preparations for inoculation mean? And how else has the death-rate in certain countries actually been so much diminished? Let us quote the *Journal of the American Medical Association* of September 12th: "There is certainly no more astonishing and gratifying fact in modern civilization than the enormous decline and still progressively decreasing death-rate of the more progressive and best governed countries. We have in England a proof of the fact which, owing to the length of time over which they extend, and the accuracy of the statistics, brings the result clearly before the mind. Estimated by quinquennial periods the English death-rate per 1,000 from 1858 to 1895 was as follows: 22.22, 22.58, 22.42, 21.96, 20.79, 19.40, 18.90, and 19,04, respectively. In London alone, the largest civilized city in the world, the rate has been reduced to about 17, while certain districts of the metropolis have reached as low a rate as 14, 13, and even 12.

"In the United States, with less concentration of population in large cities, we must confess the shameful fact that human life is more recklessly and needlessly sacrificed to the brutality of politics and sanitary heedlessness than in older countries. It is nothing less than a disgrace that in our smaller cities, and with the injuries of bad hygiene less necessary, we yet are killing off our citizens at the rate of from 5 to 10 per 1,000 faster than in European cities. Think of what this means in a city the size of Greater New York! If her death-rate is 5 per 1,000 greater than it need be, one shudders to contemplate the many thousands of citizens needlessly murdered every year.

"In some of our cities the condition makes this fact still more evident and startling. Accurate figures are not before us as we write, but we believe that in a number of moderately sized cities the death-rate has been reduced to a remarkably low figure. It has lately come to our notice that in one of our cities, Buffalo, by the persistent and heroic labors of the energetic health officer, the rate has been reduced to 11.67."

These results should surely stimulate each country and each district to vie with one another in this most useful and friendly of rivalries, and when we turn our eyes inward upon our own condition in this matter, have we reason for jubilation or the reverse? To aid us in answering that question let us inquire how the death-rate has been reduced elsewhere, and notably in England, which has been the pioneer in this good work. There, we find a "Local Government Board" with a medical department composed of a chief medical officer and subordinates, possessing the power necessary for useful action, and maintaining a certain control over the doings of the local authorities of the numerous districts into which the country is divided, and their medical officers of health. These medical officers of health in all the large towns and in the counties are paid good salaries, are not allowed to do private practice, and must give all their time to the superintendence of their inspectors, and to themselves investigating the causes of outbreaks of disease, the sanitary condition of their district, and to seeing that the health laws which have been formulated by the central office are properly observed. Reports are forwarded at regular intervals to the head of the department, who keeps up, in different ways, communications with all parts of the country.

The system of employing men who are independent of every one except the government officials, which is coming into vogue in even the most rural districts, ensures the work being done more satisfactorily than when there are patients and others to be offended. There has been for some years going on in Britain a thorough training of doctors for this service, and they are encouraged, and in nearly all cases find it not only to their advantage but necessary, to possess a special degree or diploma in public health, which is given only after a certain time spent in college and practical work in connection with the subject.

Is there anything corresponding to all this in the State of Georgia? There are boards of health, but there is no proper correspondence between the two. But there ought to be some such system in operation in Georgia. It has frequently appeared to us that, while Americans are in most respects not less careful of their rights than the inhabitants of other portions of the globe, they have shown a strange disregard for themselves in the matter of health. They have allowed less than half-fledged physicians to gain experience on their persons, and in many parts of the country they have been content to dwell in very unsavory surroundings, to an extent unworthy of such a people. Let Georgia come to the front along with some of the other States, which are bestirring themselves for their own and their country's benefit. Let there be formed in the capital city of the State a central controlling board, and let each city and each district have its own medical officer, who shall be appointed only with the consent of this board, and be ultimately responsible to it. Let all this be absolutely and entirely separate from politics, and let the medical head be a man who has had a thorough training in the subject, theoretical and practical, no matter whether he hail from South, North, East, or West; only let him be efficient, and let him be encouraged to procure properly educated subordinates. This will cost some money at the outset, but every country that has experienced its benefits will assert that it is money well spent and is in the end a financial saving. Besides, money is not everything, and good sanitation is a right of the people as much as are the facilities for good education.

We hope that this matter will not be allowed to drop. We trust that some of the more influential among the physicians of the State will take it up and, by means of meetings or otherwise, have it thoroughly ventilated, laid before the legislature, and made a subject of importance there. We doubt the ability of the legislature to see at first much more than the financial side of the question, but what is the use of medical societies if they do not let their voice be heard in matters with which they are most competent to deal?

